# Tumor Necrosis Factor α: Taking a Personalized Road in Cancer Therapy

**DOI:** 10.3389/fimmu.2022.903679

**Published:** 2022-05-18

**Authors:** Adit Ben-Baruch

**Affiliations:** The Shmunis School of Biomedicine and Cancer Research, The George S. Wise Faculty of Life Sciences, Tel Aviv University, Tel Aviv, Israel

**Keywords:** cancer, personalized therapy, tumor necrosis factor α/TNFα, TNFR1, TNFR2

## Introduction – The Long and Winding Road of TNFα in Cancer Therapy

The potent pro-inflammatory cytokine tumor necrosis factor α (TNFα) has been connected to cancer progression and treatment ever since its discovery as a major factor contributing to the anti-tumor activities of Coley’s toxins (1, 2). TNFα cloning in 1984/1985 (3, 4) and of its TNFR1 and TNFR2 receptors in 1990 (5–8) was followed by a spurt of studies demonstrating that TNFα has anti-malignancy activities. The beneficial effects of TNFα were observed *in vivo* primarily when the cytokine was administered at relatively high concentrations locally and repeatedly; the cytokine inhibited tumor growth by damaging the tumor vasculature and by directly inducing cancer cell killing, at times clearly seen when NF-κB and JNK activation was impaired (3, 9–16). Moreover, TNFα could improve the efficacy of drugs/chemotherapy in cancer treatment, e.g., by promoting blood vessel permeability (16, 17).

In clinical trials (primarily in sarcomas), TNFα was often administered locally, in order to generate high tumor concentration of the cytokine; although these trials have led to tumor regression (to various extents in the different trials), usually they did not have a pronounced ability to induce complete remission (16, 18, 19). To enable local activity of TNFα, antibody-TNFα fusion proteins (immunocytokines) were also assayed, with a relatively good efficacy in mouse cancer models, and in a small cohort of glioblastoma patients (20–23). Other cancer clinical studies have used systemic administration of TNFα, demonstrating low efficiency and giving rise to multiple side effects (15, 24, 25).

In parallel to these findings, other reports have emerged, connecting the presence of TNFα in tumors with pro-malignancy effects, and demonstrating that higher endogenous TNFα expression levels were associated with more advanced disease in cancer patients (2, 26–31). TNFα was found to be expressed in tumors from early stages of disease and on, and its continuous presence contributed to chronic inflammation, considered the Seventh Hallmark of Cancer (31–38). Moreover, the expression of TNFα by tumor cells, leukocytes and stromal cells has led to production of inflammatory chemokines that recruited leukocytes with pro-metastatic effects (26, 28, 31, 35, 38–41). Immune-suppression was also connected to TNFα presence in cancer and studies in animal models have greatly supported its tumor- and metastasis-promoting roles (2, 42–46).

Adding to these observations, numerous studies indicated that TNFα can act directly on cancer cells to promote their pro-metastatic characteristics and functions, including the generation of cancer stem cells, epithelial-to-mesenchymal transition, invasion, resistance to therapy and metabolic changes (27–31, 37, 47–51).

As research in this direction advanced, TNFα has been identified as a most powerful pro-cancer cytokine in many malignancies, suggesting that inhibitors of TNFα and/or its receptors (TNFR) could be applied in cancer treatment, alone or together with other modes of therapy.

## The Complexity of the TNFα-TNFR Network – What Is the Road Made of?

In trying to understand the opposing observations on TNFα in cancer, it is important to consider that the TNFα-TNFR network includes many different members, generating intricate interactions that are spatially and temporally regulated, leading to diverse consequences under different conditions.

Many reviews have described in detail the complexity and flexibility of the TNFα-TNFR network [e.g., (27, 52–54)]. In a nutshell, the system is identified by the following characteristics: (1) It includes a soluble and a membrane form of TNFα (mTNFα): while the receptor TNFR1 (p55) binds soluble TNFα and mTNFα, TNFR2 (p75) is fully activated by mTNFα; (2) TNFR1 is constitutively expressed by almost all cells, whereas TNFR2 expression is noted primarily in hematopoietic, endothelial and neuronal cells. (3) TNFα binding to its receptors gives rise to their trimerization, followed by unique signaling patterns of each of the receptors. Following the formation of a core signaling complex and regulation by additional intracellular components/events, TNFR1 can induce cell apoptosis and necroptosis *via* activation of its death domain; however, under different settings, TNFR1 can lead *via* activation of the NF-κB, JNK and p38 pathways to transcription of potent pro-inflammatory genes, cell survival, proliferation and motility. TNFR2, on the other hand, ultimately leads to expression of pro-inflammatory genes, cell survival and proliferation by activating canonical and non-canonical NF-κB pathways; (4) TNFR1 and TNFR2 can interact at several levels, including the ability of TNFR2 to promote the pro-apoptotic activities of TNFR1 (55, 56). (5) TNFR1 and TNFR2 have soluble forms (sTNFR1 and sTNFR2), whose activities and clinical implications are far from being fully resolved. It was suggested that at high concentrations the soluble receptors serve as sinks that reduce TNFα levels and thus inhibit its activities, while low levels of the soluble receptors enhance TNFα functions (57, 58), possibly through induction of reverse signaling following binding to mTNFα (27, 54, 59).

This very diverse array of molecular elements and events suggests that at particular settings, members of the network can lead to opposing effects. For instance, activation of TNFR1 by TNFα can lead to tumor cell death but under a different set of conditions it can contribute to cancer inflammation and enhance tumor progression. A similar enigma was observed for TNFR2+ tumor-infiltrating lymphocytes (TILs): TNFR2-mediated signals support the survival/activation of CD4+ T regulatory cells (Tregs) and aggravate disease course (46, 60–67); however, in triple-negative breast cancer (TNBC) patients, TNFR2+ TILs were associated with improved patient survival. In parallel, mouse studies have connected reduced TNBC growth after chemotherapy with elevated presence of CD8+ TNFR2+ TILs, presumably cytotoxic T cells (CTLs) (68, 69), agreeing with TNFR2 being required for cytotoxic activities of CD8+ T cells (66). Moreover, unlike several publications connecting TNFR2 expression by cancer cells to pro-tumor phenotypes (63, 70–72), TNFR2 was found to be protective in breast cancer, as demonstrated by using a mouse model with the loss of one of the TNFR2 alleles (73).

The balance and interactions between the different members of the TNFα-TNFR family – as well as their cross-talk with other factors of the TME and with different therapy modes – may dictate the path that this network takes in terms of cancer progression.

## The TNFα-TNFR Road in Cancer Therapy – The Possible Inter-Connection Of the “ THERAPY” LANE AND THE “TARGET” LANE

The information obtained so far regarding the roles of TNFα and its receptors in cancer has split the scientific and clinical communities between those who consider TNFα as “therapy” and those who regard the different members of the TNFα-TNFR family as “targets”. In practice, it is possible that these two lanes of the TNFα-TNFR road are strongly connected to each other. For example, when TNFα fails to limit metastasis in a specific setting, this may be due to selection of cytotoxicity-resistant cells that also have acquired stronger pro-metastatic functions, such as increased invasiveness or production of angiogenic factors. Moreover, many reports have demonstrated that cells treated by TNFα acquired chemoresistance, endocrine resistance and reduced sensitivity to other therapy modes (27, 30, 47, 50, 74).

These observations connect the limited therapeutic potential of TNFα to selection of cancer cells that express improved pro-metastatic functions, leading to enhanced tumor progression. Thus, treating cancer patients with TNFα may eventually give rise to devastating metastasis-promoting effects, and may prove harmful rather than beneficial.

This scenario, and the strong evidence on the pro-metastatic roles of TNFα and its receptors in many cancer types, suggest that the pro-cancer and pro-metastatic functions of the TNFα-TNFR network dominate over their protective functions in malignancy. Supporting this possibility are many studies of patients suffering of autoimmune/inflammatory diseases, demonstrating that inhibition of the TNFα-TNFR pathway was not significantly associated with increased tumor risk or recurrence (with some, yet unsubstantiated, reservations regarding non-melanoma skin cancer and lymphoma) (75–81).

Taken together, the findings obtained so far suggest that when the TNFα-TNFR network is considered in cancer therapy, the “target” approach may apply better than the “therapy” tactic. Yet, to date, only a very limited number of clinical studies had analyzed the therapeutic value of TNFα-TNFR antagonists in cancer treatment. In several phase I and phase II trials, patients at locally advanced or metastatic stages of different malignant diseases were treated by antibodies against TNFα (infliximab) or soluble TNFR2 (etanercept). Partial or complete responses were noted only in a renal cell carcinoma study, but disease stabilization was observed in some of the patients in the other studies (82–85). In addition, a recent phase Ib clinical trial demonstrated relatively high response rates following the use of the TNFα inhibitor certolizumab together with anti-PD-1 and anti-CTLA-4 in melanoma patients (86).

Overall, as these clinical trials have been performed under unfavorable conditions – the cohort patients were at the most advanced stages of disease, and their immune system has been already manipulated by repeated therapies – their findings suggest that TNFα-TNFR-directed treatments may be effective in cancer. It is possible that if inhibitors of the TNFα-TNFR family members will be given the most optimal conditions to act, and if the targets will be carefully selected, better therapeutic indices could be achieved.

## Discussion – The (Personalized) Road Ahead

To reach the aim of safe and effective use of TNFα-TNFR manipulations in cancer therapy, we need to consider the possibility that one type of TNFα-TNFR-directed therapy is not suitable to all cancer types and to all cancer patients; moreover, a specific therapy mode that applies to one cancer type/subtype may be detrimental in another.

Rather, the typical characteristics of tumor heterogeneity – inter-tumor and intra-tumor – call for a personalized approach that will carefully adjust the therapy mode and the treatment conditions to each malignancy type. First, it may be important to pre-select the patients who will most probably benefit from the modulation of TNFα-TNFR family members, and to start therapy as early as possible, to prevent the pro-metastatic activities of the network. For example, favorable candidates for treatment may be patients diagnosed at the early stage of breast ductal carcinoma *in situ*, whose tumors express TNFα (about half of the patients) (31).

Then, the roles of each family member should be precisely identified in each cancer type/subtype, prior to treating patients with modulators of the pathway. This can be well-exemplified by taking the TNFR2+ TIL population as a test case: the fact that unlike published reports on the Treg identify of CD4+ TNFR2+ lymphocytes (46, 60–67), TNFR2+ TILs were connected to improved survival in TNBC patients and with potential cytotoxic activities of CD8+ TNFR2+ TILs in mouse TNBC tumors (68, 69), suggests that targeting TNFR2 in chemotherapy-treated TNBC patients may be harmful; administration of TNFα inhibitors may reduce the proliferation of CD8+ TNFR2+ CTLs and limit the potential of raising potent immune activities against the cancer cells. The detrimental consequence that may be driven by such treatments may explain the findings obtained in TNFα-/- mice that could not mount T cell-mediated anti-tumor effects (87). Rather, the use of TNFR2 agonists (53, 54) may apply in order to promote the proliferation of cytotoxic CD8+ TNFR2+ TILs; alternatively, selective inhibitors of TNFR1 [(once clinically-approved (53, 54)] may be best suited in therapy as they may limit the chronic inflammation that is strongly induced by TNFα in the tumors.

**Figure 1:**
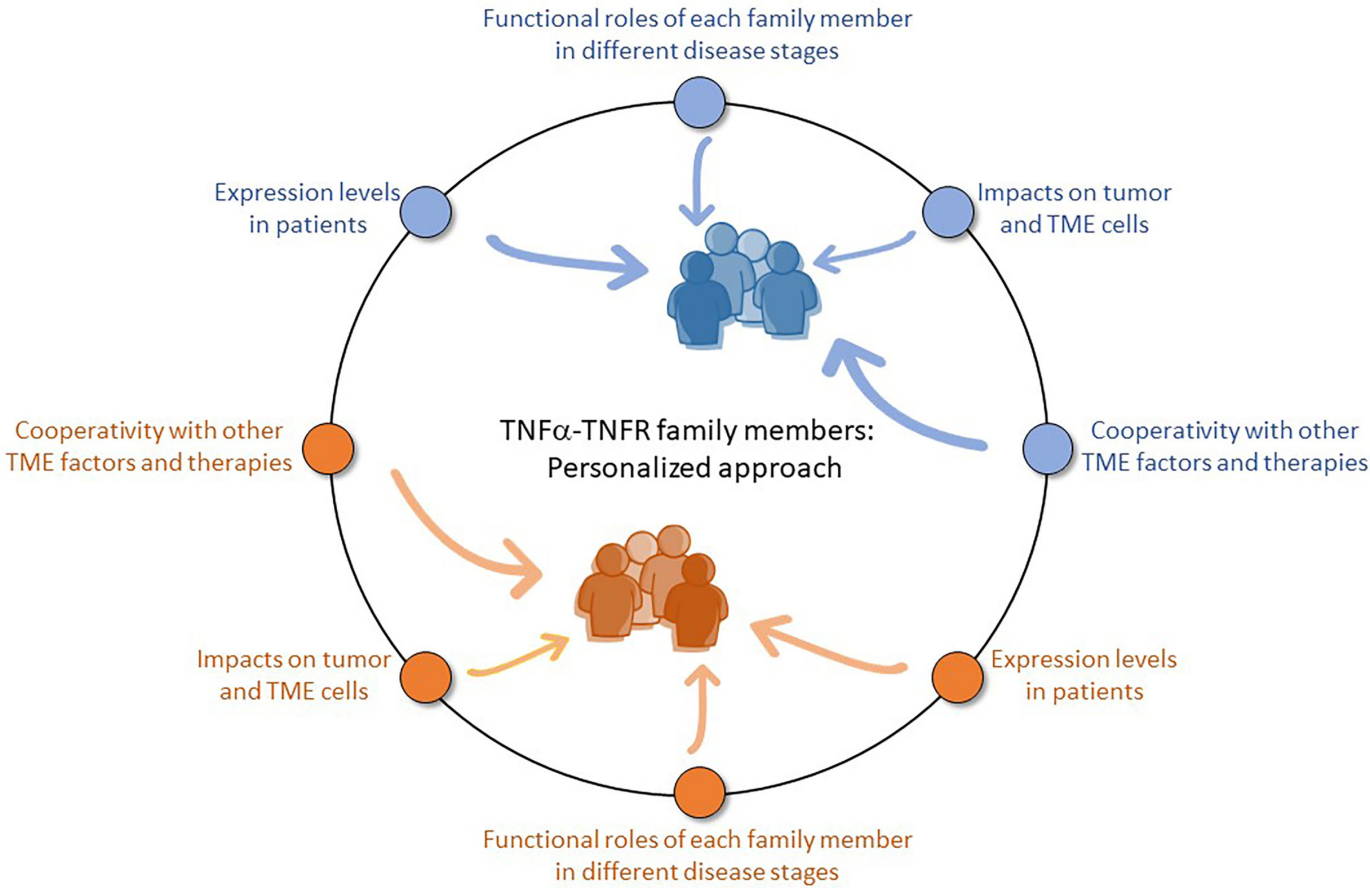
Targeting members of the TNFα-TNFR family in cancer: The personalized road. The TNFα-TNFR network brings together complex interactions between the soluble and membrane forms of TNFα, as well as TNFR1 and TNFR2 with their diverse binding preferences to each of the TNFα forms, complex signaling cascades and soluble variants. This intricate system of ligands and receptors can lead to different consequences in various malignancies, raising the need to carefully identify specific players in each and every disease type, and possibly also in patients who were diagnosed with the same type of cancer. Thus, a personalized approach should be designed in order to establish the most appropriate and efficient therapeutic mode in cancer, vis-à-vis the use of modifiers of TNFα and its receptors. For example, the properties of one malignant disease (“Blue patients” in the Figure) in terms of TNFα-TNFR family members may considerably differ from the characteristics of another cancer type (“Orange patients”). To precisely identify who can benefit from treatments directed to any of the family members, it is necessary to determine the expression patterns of each partner in each patient; then, based on analyses of each malignancy and its subtypes, it is necessary to determine their roles in different disease stages, their impacts on different cell types in the tumor (cancer cells, endothelial cells, leukocytes, stromal cells), as well as their interactions with other TME factors and potential efficacy when combined with other cancer therapies. The information obtained by research of the different aspects and elements stands in the basis of a personalized approach that will target members of the TNFα-TNFR family or exploit them (e.g., by increasing the proportions/activities of beneficial TNFR2+ CD8+ TILs) that would reach an outmost efficacy in cancer therapy.

Thus, when manipulation of members of the TNFα-TNFR family is considered in cancer therapy, one needs to determine many aspects in a most specific manner (**Figure 1**): who are the patients who can benefit from the treatment? Which TNFα-TNFR family member – in its membrane or soluble form – or its down-stream intracellular regulators, should be targeted? Do the various network members affect similarly different types of cells in a specific tumor type/subtype: cancer cells, endothelial cells, leukocytes and others? How TNFα activities are affected by other factors of the TME and regulate them [e.g., estrogen, EGF and TGFβ (30, 49, 50, 88, 89)]? Can TNFα-TNFR-directed therapies reach improved impacts when administered with other treatments, as reported recently to be the case with immune checkpoint blockades (86, 90, 91)?

To conclude, the two seemingly opposing effects of the TNFα-TNFR network on cancer progression may be actually inter-connected, and eventually the pro-metastatic functions of the TNFα-TNFR family members possibly dominate their anti-malignancy effects. Moreover, the “one therapy mode suits all” approach in targeting the TNFα-TNFR pathway in cancer needs to be re-evaluated, and emphasis should be given to extensive research that will identify the most appropriate therapeutic mode for each malignancy type/subtype, in a specific and personalized manner.

## Author Contributions

AB has designed the article, drafted all versions and is responsible for its contents.

## Funding

Studies in the Ben-Baruch laboratory on the TNFα-TNFR network in cancer were supported during the last three years by DKFZ-MOST Cooperation in Cancer Research, Israel Cancer Research Fund, Federico Foundation and Israel Science Foundation.

## Conflict of Interest

The author declares that the research was conducted in the absence of any commercial or financial relationships that could be construed as a potential conflict of interest.

## Publisher’s Note

All claims expressed in this article are solely those of the authors and do not necessarily represent those of their affiliated organizations, or those of the publisher, the editors and the reviewers. Any product that may be evaluated in this article, or claim that may be made by its manufacturer, is not guaranteed or endorsed by the publisher.
